# Development and Validation of a Novel Prognostic Model for Acute Myeloid Leukemia Based on Immune-Related Genes

**DOI:** 10.3389/fimmu.2021.639634

**Published:** 2021-05-05

**Authors:** Ran Li, Zuoyou Ding, Peng Jin, Shishuang Wu, Ge Jiang, Rufang Xiang, Wenfang Wang, Zhen Jin, Xiaoyang Li, Kai Xue, Xiaolu Wu, Junmin Li

**Affiliations:** ^1^ Shanghai Institute of Hematology, State Key Laboratory of Medical Genomics, National Research Center for Translational Medicine at Shanghai, Ruijin Hospital Affiliated to Shanghai Jiao Tong University School of Medicine, Shanghai, China; ^2^ Department of General Surgery, Zhongshan Hospital of Fudan University, Shanghai, China; ^3^ Department of Children Health Care, Women’s Hospital of Nanjing Medical University, Nanjing Maternity and Child Health Care Hospital, Nanjing, China

**Keywords:** acute myeloid leukemia, prognostic signature, immune-relate genes, The Cancer Genome Atlas, Least Absolute Shrinkage and Selection Operator

## Abstract

The prognosis of acute myeloid leukemia (AML) is closely related to immune response changes. Further exploration of the pathobiology of AML focusing on immune-related genes would contribute to the development of more advanced evaluation and treatment strategies. In this study, we established a novel immune-17 signature based on transcriptome data from The Cancer Genome Atlas (TCGA) and The Genotype-Tissue Expression (GTEx) databases. We found that immune biology processes and transcriptional dysregulations are critical factors in the development of AML through enrichment analyses. We also formulated a prognostic model to predict the overall survival of AML patients by using LASSO (Least Absolute Shrinkage and Selection Operator) regression analysis. Furthermore, we incorporated the immune-17 signature to improve the prognostic accuracy of the ELN2017 risk stratification system. We concluded that the immune-17 signature represents a novel useful model for evaluating AML survival outcomes and may be implemented to optimize treatment selection in the next future.

## Introduction

Acute myeloid leukemia (AML) is one of the most common hematological cancers in adults, characterized by the accumulation of immature myeloblasts in the bone marrow and peripheral blood at the expense of normal blood components (1). Unlike many other cancers, AML has a low tumor mutation burden (TMB) with an average of 10–13 coding mutations per patient (2). Although we have understood the role of mutational genes in driving tumor progression along with an uncomplicated mutational landscape, the overall therapeutic strategy for AML patients has remained the same for the last 30 years (3, 4). The conventional treatment paradigm has a restricted contribution to improve overall survival (OS), especially in the elderly population (4). Although allogeneic hematopoietic cell transplantation (alloHCT) and chemotherapy regimens allowed a five-year survival rate of 40–70% in younger patients (<40 years of age), survival in the elderly remains poor (5, 6) with a risk of relapse within 5 years from diagnosis as high as 75% (7). Therefore, it is urgent to identify potential biomarkers to inform prognosis and treatment allocation in this setting.

It has been well proved that allogeneic hematopoietic cell transplantation (alloHCT) is successful in treating AML and AML is immune-responsive (8). However, immunosuppression could also be caused by AML blasts, leading to paradoxical immunosuppression in patients with AML (9). Therefore, further understanding of how immune cells battle with AML blasts could lead to more effective therapies for AML.

Previous studies (10–12) have stratified AML patients into low- and high-risk groups based on immune and stromal scores with the ESTIMATE algorithm (13), which is based on single sample Gene Set Enrichment Analysis and then generates stromal and immune scores to predict the infiltration of stromal and immune cells in tumors. The identified differentially expressed genes (DEGs) from low- and high-risk groups were then studied to look for potential prognostic value in AML patients. However, there is a lack of a comprehensive study on the utility of immune-related gene (IRG) expression in predicting AML prognosis and comparing AML with healthy samples. This study aimed to fill the gap by focusing on the relationship between IRG expression and AML patients’ prognosis.

## Materials and Methods

### Data Acquisition

Clinical and transcriptome information of TCGA-LAML and GTEx-whole blood cohorts were acquired from the UCSC Xena database (http://xena.ucsc.edu/). The GTEx project is a data resource of the healthy population from organ donation and rapid autopsy settings (14). All the transcriptome data have been normalized according to the description from the UCSC Xena database. The ImmPort database provided a total of 2,498 immune-related genes. We obtained clinical and transcriptome data from the GSE37642 cohort (validation cohort) in the Gene Expression Omnibus (GEO) database to validate our prognostic model. All eligible samples from TCGA and validation cohorts were collected according to the following inclusive criteria: (1) newly diagnosed acute myeloid leukemia specimen; (2) Availability of transcriptome data; (3) Availability of general survival information and related clinical data. The clinical information of inclusive AML samples is detailed in **Table 1**.

**Table 1:** Baseline characteristics of the patients in the training and validation cohorts.

Clinicopathological variables	Training dataset (n = 151)			Clinicopathological variables	Validation dataset (n = 417)		
	high-risk group	low-risk group	p		high-risk group	low-risk group	p
**Age (years)**			0.004	**Age (years)**			0.256
<60	33	51		<60	108	119	
≥60	42	25		≥60	101	89	
**FAB classification**			0.01	**FAB classification**			0.003
M0	10	5		M0	11	3	
M1	20	15		M1	40	44	
M2	15	23		M2	49	68	
M3	4	11		M3	4	15	
M4	10	19		M4	57	47	
M5	12	3		M5	30	17	
M6	2	0		M6	7	8	
M7	1	0		M7	2	0	
**Gender**			0.467	**Status**			0.001
Female	36	32		Alive	40	69	
Male	39	44		Dead	169	139	
**Status**			<0.0001	**RUNX1-RUNX1T1**			0.001
Alive	11	43		Negative	205	189	
Dead	64	33		Positive	4	19	
**WBC**			0.787	**RUNX1 mutation**			0.043
<10 × 10^9^/L	28	30		Negative	153	157	
≥10 × 10^9^/L	47	46		Positive	37	21	
**BM blast**			0.559				
<70%	31	35					
≥70%	44	41					
**Risk (Cytogenetic)**			<0.0001				
Good	7	24					
Intermediate	43	38					
Poor	24	12					
**ELN2017**			<0.0001				
Favorable	7	25					
Intermediate	28	23					
Adverse	36	17					
**Transplant**			0.161				
Yes	29	38					
No	46	38					
**Chemotherapy**			0.638				
Yes	72	74					
No	3	2					
**Relapse**			0.12				
Yes	38	29					
No	36	46					

FAB, French-American-British; WBC, white blood cell; BM, bone marrow; ELN, European LeukemiaNet.

### Establishment of an Immune-Related Signature

2,516 DEGs were identified between the TCGA-LAML and GTEx-whole blood cohorts (**Table S1**). After integrating 2,498 IRGs, we obtained 199 differentially expressed IRGs (**Table S2**). Univariate Cox regression analysis was performed to evaluate the association between expression levels of individual IRGs and OS and 72 of them were of potential prognostic value (**Table S3**). To minimize the risk of overfitting, LASSO regression analysis was applied to construct a prognostic model (15). We finally established an immune prognostic signature (immune-17 signature, because it contains 17 IRGs). The risk score was calculated using the equation: β1 × gene1 expression + β2 × gene2 expression + … + βn × genen expression, where β was the correlation coefficient generated by LASSO regression analysis.

### Evaluation of the Immune-17 Signature

Each patient from the GEO or TCGA database was allocated a risk score derived from the immune-17 signature. These patients were then stratified into low- and high-risk groups using the median risk score as the cutoff value. The Kaplan–Meier analysis was conducted to evaluate the prognostic significance of the immune-17 signature. Model specificity and sensitivity were assessed by calculating the area under the curve (AUC) values. Specific predictive ability was determined when AUC >0.60, while excellent predictive values were determined if AUC >0.75. Univariate and multivariate Cox analysis were used to prove the signature is an independent prognostic model.

### Improvement of European LeukemiaNet (ELN) 2017 Risk Stratification System

Patients were stratified into three new groups: ELN favorable/immune-17^high^ and ELN adverse/immune-17^low^ patients were re-assigned to the intermediate-risk group, and ELN intermediate/immune-17^high^ patients were re-assigned to the high-risk group. Through Kaplan–Meier analysis, we evaluated the prognostic significance of the new risk stratification system.

### Statistical Analysis

The R software (version 4.0.2, https://www.r-project.org/) was used to perform all statistical analyses. DEGs between healthy individuals and AML patients were identified using the “limma” package with filter criteria (FDR <0.05 and |log FC |>2). Heatmap and clustering were carried out using the “pheatmap” package. Enrichment analysis was performed using the “clusterProfiler” package. Univariate and multivariate Cox regression analysis was conducted using “survival” package. “glmnet” and “survival” packages were used to conduct LASSO regression analysis. “survminer” and “survival” packages were used to perform Kaplan–Meier analysis. “survivalROC” package was used to determine AUC values and construct receiver operating characteristic (ROC) curves. The introduction of packages in R software can be found at the site of (https://cloud.r-project.org/). All statistical tests were two-sided, and p <0.05 was considered to be statistically significant.

## Results

### IRGs Were Associated With the OS of AML Patients

**Figure 1:**
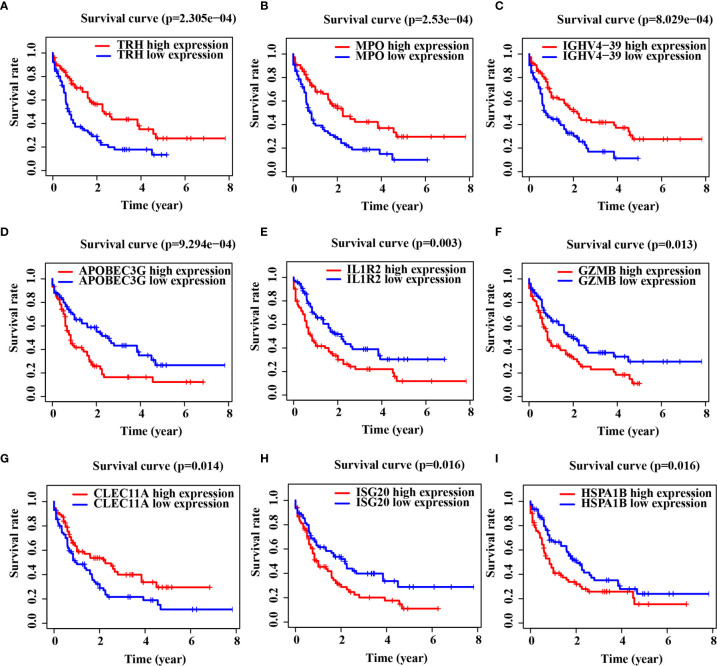
Immune-related genes (IRGs) associated with the overall survival (OS) of acute myeloid leukemia (AML) patients. **(A–I)** Kaplan–Meier curve analysis of nine representative IRGs in TCGA-LAML cohort.

Gene Ontology (GO) and Kyoto Encyclopedia of Genes and Genomes (KEGG) pathway enrichment analyses indicated that DEGs were mainly enriched in the immune biology processes (**Figure S1A**). The KEGG enrichment analysis results revealed the central role of transcriptional dysregulations in the development of AML (**Figure S1B**). 17 IRGs involved in the model were associated with the OS of AML patients (**Table 2**), and their expression in AML patients is shown in **Table S2**. Representative Kaplan–Meier plots showed TRH, MPO, IGHV4-39 and CLEC11A associated with prolonged survival of AML patients. On the contrary, APOBEC3G, IL1R2, GZMB, ISG20 and HSPA1B correlated with a poor OS (**Figures 1A–I**).

**Table 2:** Univariate Cox regression analysis of 17 genes from immune-17 model for overall survival of TCGA-LAML patients.

id	HR	HR.95L	HR.95H	p-value	Coef
CALR	0.5621	0.4192	0.7537	0.0001	−0.0376
HSPA1B	1.2640	1.0710	1.4918	0.0056	0.0362
APOBEC3G	1.8348	1.3200	2.5503	0.0003	0.0589
MX1	1.2173	1.0787	1.3736	0.0014	0.0687
ISG20	1.6087	1.2889	2.0077	0.0000	0.1710
MPO	0.8764	0.8274	0.9284	0.0000	−0.0483
CCL4	1.3606	1.0863	1.7041	0.0074	0.1340
FGR	1.1088	1.0253	1.1991	0.0097	0.0032
MIF	1.2278	1.0085	1.4948	0.0410	0.2294
IGHD5.18	1.1075	1.0345	1.1856	0.0033	0.0108
IGHV4.39	0.8980	0.8301	0.9714	0.0073	−0.0794
IGHV5.51	0.8796	0.8042	0.9620	0.0050	−0.0608
PLXNB2	1.2653	1.0672	1.5002	0.0068	0.0676
CLEC11A	0.8809	0.8211	0.9450	0.0004	−0.0324
TRH	0.8622	0.7922	0.9385	0.0006	−0.0645
IL1R2	1.1756	1.0602	1.3035	0.0021	0.0939
GZMB	1.3243	1.1444	1.5324	0.0002	0.0535

HR (Hazard ratio) is intended for overall survival; Coef, correlation coefficient.

### Evaluation of the IRG Signature

**Figure 2:**
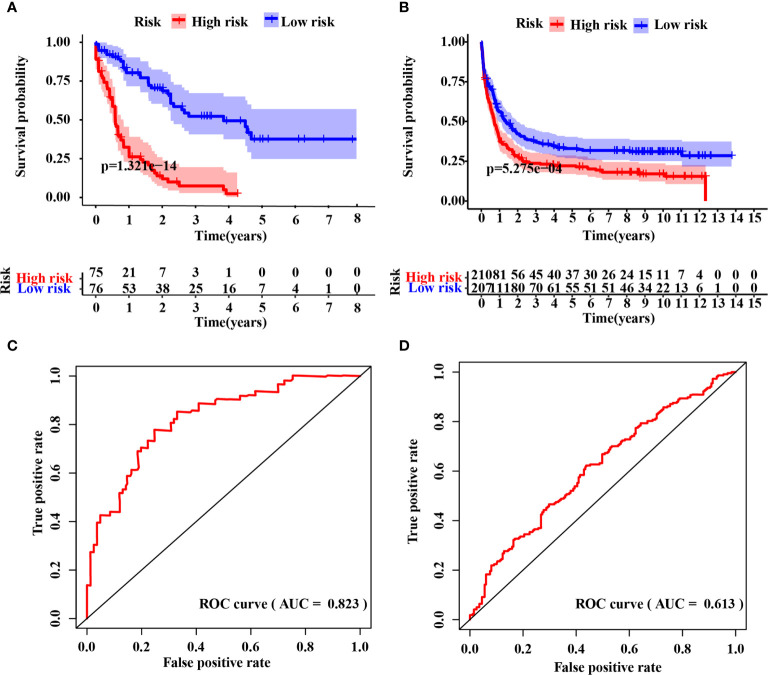
Evaluation of the IRG signature. Kaplan–Meier curve analysis between the high- and low-risk groups in the raining **(A)** and validation **(B)** cohort, respectively. Time-dependent receiver operating characteristic (ROC) curve analysis of the immune-17 signature in the training **(C)** and validation **(D)** cohort, respectively.

**Figure 3:**
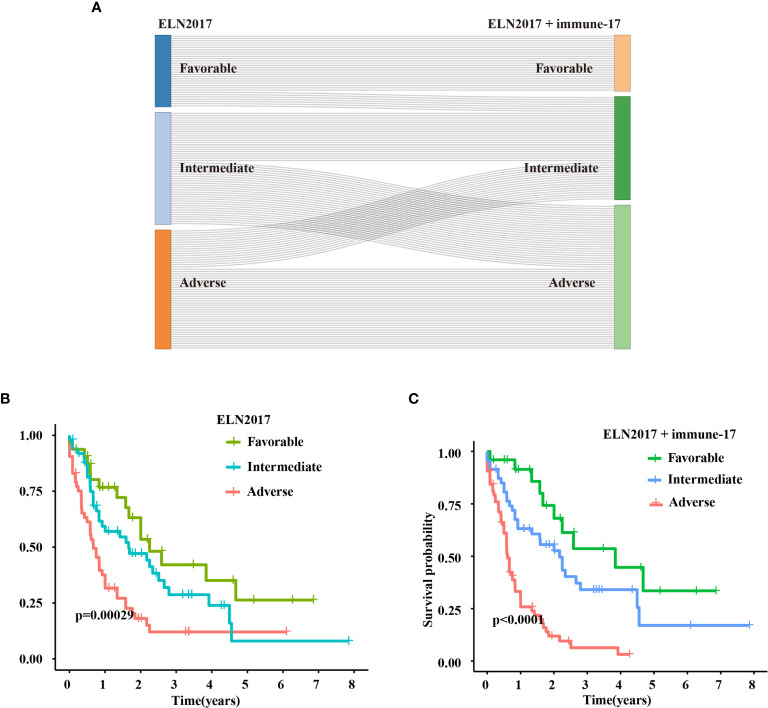
Improved ELN2017 risk stratification system. **(A)** Re-stratification of patients from the three ELN2017 categories to the novel three ELN2017+immune-17 categories. Each line represents a patient. The line’s left end means the ELN2017 categories and the line’s right end means novel ELN-immune-17 categories. If the line is parallel, the patient’s classification is not change. If not, the patient’s classification has changed. Kaplan–Meier analysis for AML patients stratified by ELN2017 risk stratification system **(B)** or ELN2017+immune-17 risk stratification system **(C)**.

The IRG signature was evaluated in the training (TCGA-LAML) and validation cohorts. Kaplan–Meier plots demonstrated that patients allocated to the high-risk group showed a significantly shorter OS (p = 1.321e−14, TCGA-LAML; p = 5.275e−4, validation cohort), (**Figures 2A, B**). This model’s AUC value achieved a value of 0.823 in the TCGA-LAML cohort and a value of 0.613 in the validation cohort, respectively (**Figures 2C, D**). To determine whether the immune-17 signature was a stable predictive model, we performed the Kaplan-Meier analysis in patients from the TCGA-LAML cohort stratified by age (**Figure S2A**) and ELN risk system (**Figure S2B**), respectively. The results indicated that the immune-17 signature could discern low-risk patients from high-risk patients in all stratified groups. We performed the univariate and multivariate Cox analysis revealed that the signature was an independent prognostic model (univariate Cox: hazard ratio [HR], 1.768; 95% confidence interval [95% CI], 1.559–2.019; p <0.001; multivariate Cox: HR, 1.631; 95% CI, 1.417–1.878; p <0.001), (**Table 3**). After re-stratification of patients (**Figures 3A**), we found that combined ELN + immune-17 risk stratification system could more accurately define AML patients’ prognosis (**Figures 3B, C**).

**Table 3:** Univariate and multivariate Cox regression analysis of immune-17 model for overall survival of TCGA-LAML patients.

Characteristics	Univariate Cox				Multivariate Cox			
	HR	HR.95L	HR.95H	p-value	HR	HR.95L	HR.95H	p-value
**Age**	1.0324	1.0171	1.0479	2.73E−05	1.0258	1.0102	1.0416	0.0011
**Sex**	1.0141	0.6684	1.5386	0.9474	0.7646	0.4824	1.2118	0.2533
**BM Blast (%)**	1.0025	0.9920	1.0131	0.6431	1.0058	0.9944	1.0172	0.3201
**WBC**	1.0019	0.9972	1.0067	0.4242	1.0034	0.9984	1.0083	0.1824
**Platelet count**	0.9999	0.9964	1.0034	0.9408	0.9983	0.9943	1.0024	0.4199
**ELN2017**	1.7708	1.3287	2.3600	9.64E−05	1.6412	1.1859	2.2711	0.0028
**Immune-17 score**	1.7685	1.5490	2.0190	3.35E−17	1.6313	1.4167	1.8784	1.04E−11

HR is intended for overall survival; WBC, white blood cell; BM, bone marrow; ELN, European LeukemiaNet.

## Discussion

Genetic and clinical factors play increasingly important roles in predicting OS and event-free survival (EFS) for AML patients (16). Patient-related factors, AML-related genetic factors and MRD monitoring are considered as key factors responsible for AML prognosis (17). In the past, immune factors have been largely ignored. This study constructed an immune prognostic signature for AML based on the TCGA-LAML and GTEx-whole blood cohort, which improved the accuracy of ELN2017 risk stratification system.

Among the 17 IRGs involved in the immune-17 signature, we roughly divided them into three categories according to genes’ function: 1) innate immunity-related genes; 2) specific immunity-related genes; 3) endocrine-related genes. APOBEC3G, MIF, MX1, ISG20, MPO, FGR, and IL1R2 are innate immunity-related genes. APOBEC3G is highly expressed in various cancers and plays an essential role in regulating tumor growth and innate immune responses (18, 19). MIF contributes to the immune escape, anti-inflammatory, and immune tolerance in either innate or adaptive immune cells (20). Primary AML highly expressed MIF and MIF drives the bone marrow mesenchymal stromal cells (BM-MSC) to express IL-8, which in turn assists AML cell survival and proliferation (21). Base on this finding, an anti-MIF monoclonal antibody (Imalumab) is being studied in a phase I study (NCT01765790) to assess the safety, pharmacokinetics, tolerability, and antitumor activity against solid cancers (22). FGR is a member of the Src family and contributes to the transition of signals from cell surface receptors and promotes inflammatory cytokines releases. FGR expression is restricted to myeloid lineage and is markedly highly expressed in a subset of AML (23). CALR, CCL4, and GZMB are considered genes of the adaptive immunity. CALR is found in myeloproliferative neoplasms (MPN) and represents an MPN-driver mutation. The presence of this gene is included in the current diagnostic criteria of Ph^(−)^ MPN (24). The AML patients converted from MPN had more CALR mutation rate frequency (25). Moreover, there are reports that the expression of CALR is remarkably higher than other hematologic malignancies, such as ambiguous lineage, ALL, MPN, MDS/MPN (26). CCL4 and GZMB are associated with T-cell immunity. CCL4 is a biomarker of multiple sclerosis and associated with inflammation and T-cell activation (27). AML patients with monocytic differentiation had increased serum levels of CCL4 along with CCL5 and CCL3. The three biomarkers promote an inflammatory state and participated in the progression of suppressing T cell-related immune response (28). GZMB encodes the preprotein secreted by NK cell and CTLs, which is related to the apoptosis of target cells (29). Hypermethylation of the enhancer upstream of GZMB might contribute to an inferior overall survival of AML (30). The mutation in these genes might cause obstacles to the elimination of abnormal cells. Finally, TRH and CLEC11A not only have functions in the endocrine system, but also play a role in immune system. Other genes included in the 17-immune signature have been reported to participate in immune response. These include HSPA1B, IGHD5-18, IGHV4-39, IGHV5-51, and PLXNB2, providing directions and clues for our future research.

Although the immune-17 signature has been proved effective in both training and validation cohorts, the AUC value for this model applied in the validation cohorts is not satisfying; it might be due to different data platforms. The training cohort is generated from RNA-sequencing, while the validation cohort is obtained from the microarray platform. In addition, the different ethnicity distribution may contribute to this result. Although the AUC value is lower in the validation cohort than that in the training cohort, the signature still exhibited predictive power in validation cohort evidenced by the Kaplan–Meier survival and ROC curve analysis.

ELN2017 risk stratification system (integrated cytogenetic and mutational status information) has been utilized in general practice (17). However, non-genetic potential mechanisms have been found to play essential roles in AML patients’ survival (31, 32). In this study, from the immunology perspective, we improved the accuracy of ELN2017 risk stratification system by incorporating the immune-17 signature. Different immune signatures in myeloid neoplasms need to be investigated, which will likely improve knowledge on disease pathogenesis and inform novel therapies. Several novel drugs such as anti-TIM3 and anti-CD47 antibodies are macrophage and lymphocyte immune checkpoint inhibitors, which are under active investigation and (33, 34). These drugs reactivate immune response against myeloid blasts, thus blocking leukemia immune escape.

In conclusion, we constructed an immune-related signature, which is a reliable and accurate model to predict AML patients’ OS. We also refined the ELN2017 risk stratification system after the incorporation of this model. The immune-17 signature may be implemented to refine AML prognosis and, in the future, to inform treatment with novel immunotherapies.

## Data Availability Statement

The datasets presented in this study can be found in online repositories. The names of the repository/repositories and accession number(s) can be found below: (https://www.ncbi.nlm.nih.gov/), GSE37642.

## Author Contributions

RL, ZD, and PJ performed the research. RL designed the research study. SW, GJ, RX, WW, ZJ, and XL analyzed the data. RL, KX, XW, JL and PZ wrote and revised the paper. All authors contributed to the article and approved the submitted version.

## Funding

This work was supported by the National Key Research and Development Program of China (2019YFA0905900), the Science and technology development fund project of Nanjing Medical University (NMUB2019224) and the Scientific Research Project of Shanghai Science and Technology Committee (17411952000).

## Conflict of Interest

The authors declare that the research was conducted in the absence of any commercial or financial relationships that could be construed as a potential conflict of interest.

## Supplementary Material

Supplementary Figure 1 Enrichment analysis of differentially expressed genes (DEGs) derived from AML patients and healthy individuals. **(A)** The top 10 Gene Ontology (GO) terms of biological process. **(B)** The top 5 Kyoto Encyclopedia of Genes and Genomes (KEGG) terms.

Supplementary Figure 2 Stratification analysis. Kaplan-Meier analysis of AML patients from TCGA-LAML cohort stratified by age **(A)** and ELN risk stratification system **(B)**.

Supplementary Table 1 2516 DEGs between AML patients and healthy individuals.

Supplementary Table 2 199 differentially expressed IRGs between AML patients and healthy individuals.

Supplementary Table 3 Univariate Cox analysis of 199 differentially expressed IRGs.

## References

[1] Khwaja A Bjorkholm M Gale RE Levine RL Jordan CT Ehninger G. acute Myeloid Leukaemia. Nat Rev Dis Primers (2016) 2:16010. 10.1038/nrdp.2016.10 27159408

[2] Ley TJ Miller C Ding L Raphael BJ Mungall AJ Robertson A. Genomic and Epigenomic Landscapes of Adult De Novo Acute Myeloid Leukemia. New Engl J Med (2013) 368(22):2059–74. 10.1056/NEJMoa1301689 PMC3767041 23634996

[3] Coombs CC Tallman MS Levine RL. Molecular Therapy for Acute Myeloid Leukaemia. Nat Rev Clin Oncol (2016) 13(5):305–18. 10.1038/nrclinonc.2015.210 PMC5525060 26620272

[4] Döhner H Weisdorf DJ Bloomfield CD. Acute Myeloid Leukemia. New Engl J Med (2015) 373(12):1136–52. 10.1056/NEJMra1406184 26376137

[5] Shah A Andersson TM Rachet B Björkholm M Lambert PC. Survival and Cure of Acute Myeloid Leukaemia in England, 1971-2006: A Population-Based Study. Br J Hematol (2013) 162(4):509–16. 10.1111/bjh.12425 23786647

[6] Andersson TM Lambert PC Derolf AR Kristinsson SY Eloranta S Landgren O. Temporal Trends in the Proportion Cured Among Adults Diagnosed With Acute Myeloid Leukaemia in Sweden 1973-2001, a Population-Based Study. Br J Hematol (2010) 148(6):918–24. 10.1111/j.1365-2141.2009.08026.x 19995394

[7] van Galen P Hovestadt V Wadsworth Ii MH Hughes TK Griffin GK Battaglia S. Single-Cell Rna-Seq Reveals Aml Hierarchies Relevant to Disease Progression and Immunity. Cell (2019) 176(6):1265–81.e1224. 10.1016/j.cell.2019.01.031 30827681 PMC6515904

[8] Beyar-Katz O Gill SJ. Novel Approaches to Acute Myeloid Leukemia Immunotherapy. Clin Cancer Res (2018) 24(22):5502–15. 10.1158/1078-0432.CCR-17-3016 29903894

[9] Ustun C Miller J Munn D Weisdorf D Blazar BJB. Regulatory T Cells in Acute Myelogenous Leukemia: Is it Time for Immunomodulation? Blood (2011) 118: (19):5084–95. 10.1182/blood-2011-07-365817 PMC3217399 21881045

[10] Huang S Zhang B Fan W Zhao Q Yang L Xin W. Identification of Prognostic Genes in the Acute Myeloid Leukemia Microenvironment. Aging (2019) 11(22):10557–80. 10.18632/aging.102477 PMC6914404 31740623

[11] Yan H Qu J Cao W Liu Y Zheng G Zhang E. Identification of Prognostic Genes in the Acute Myeloid Leukemia Immune Microenvironment Based on TCGA Data Analysis. Cancer Immunol Immunother CII (2019) 68(12):1971–8. 10.1007/s00262-019-02408-7 PMC11028253 31650199

[12] Wang S Yang L Liu Y Xu Y Zhang D Jiang Z. A Novel Immune-Related Competing Endogenous RNA Network Predicts Prognosis of Acute Myeloid Leukemia. Front Oncol (2020) 10:1579. 10.3389/fonc.2020.01579 32850463 PMC7432272

[13] Yoshihara K Shahmoradgoli M Martínez E Vegesna R Kim H Torres-Garcia W. Inferring Tumour Purity and Stromal and Immune Cell Admixture From Expression Data. Nat Commun (2013) 4:2612. 10.1038/ncomms3612 24113773 PMC3826632

[14] Aguet F Anand S Ardlie KG Gabriel GA Getz H Graubert A. The GTEx Consortium Atlas of Genetic Regulatory Effects Across Human Tissues. Sci (New York NY) (2020) 369(6509):1318–30. 10.1126/science.aaz1776 PMC7737656 32913098

[15] Friedman J Hastie T Tibshirani R. Regularization Paths for Generalized Linear Models Via Coordinate Descent. J Stat Softw (2010) 33(1):1–22. 10.18637/jss.v033.i01 20808728 PMC2929880

[16] Short NJ Rytting ME Cortes JE. Acute Myeloid Leukaemia. Lancet (London England) (2018) 392(10147):593–606. 10.1016/S0140-6736(18)31041-9 PMC10230947 30078459

[17] Döhner H Estey E Grimwade D Amadori S Appelbaum FR Büchner T. Diagnosis and Management of AML in Adults: 2017 ELN Recommendations From an International Expert Panel. Blood (2017) 129(4):424–47. 10.1182/blood-2016-08-733196 PMC5291965 27895058

[18] Okada A Iwatani YJ. Apobec3g-Mediated G-to-A Hypermutation of the HIV-1 Genome: The Missing Link in Antiviral Molecular Mechanisms. Front Microbiol (2016) 7:2027. 10.3389/fmicb.2016.02027 28066353 PMC5165236

[19] Garg A Kaul D NJ C. bc, Molecules, Diseases: APOBEC3G Governs to Ensure Cellular Oncogenic Transformation. Blood Cells Mol Dis (2015) 55: (3):248–54. 10.1016/j.bcmd.2015.07.009 26227855

[20] Noe JT Mitchell RA. Mif-Dependent Control of Tumor Immunity. Front Immunol (2020) 11:609948. 10.3389/fimmu.2020.609948 33324425 PMC7724107

[21] Abdul-Aziz AM Shafat MS Mehta TK Di Palma F Lawes MJ Rushworth SA. Mif-Induced Stromal Pkcβ/Il8 Is Essential in Human Acute Myeloid Leukemia. Cancer Res (2017) 77(2):303–11. 10.1158/0008-5472.CAN-16-1095 27872094

[22] Mahalingam D Patel MR Sachdev JC Hart LL Halama N Ramanathan RK. Phase I Study of Imalumab (BAX69), a Fully Human Recombinant Antioxidized Macrophage Migration Inhibitory Factor Antibody in Advanced Solid Tumours. Br J Clin Pharmacol (2020) 86(9):1836–48. 10.1111/bcp.14289 PMC7444762 32207164

[23] Shen K Moroco JA Patel RK Shi H Engen JR Dorman HR. The Src Family Kinase Fgr is a Transforming Oncoprotein That Functions Independently of SH3-SH2 Domain Regulation. Sci Signaling (2018) 11(553). 10.1126/scisignal.aat5916 30352950

[24] Ok C Trowell K Parker K Moser K Weinberg O Rogers H. Chronic Myeloid Neoplasms Harboring Concomitant Mutations in Myeloproliferative Neoplasm Driver Genes (JAK2/MPL/CALR) and SF3B1. Mod Pathol (2020) 34:20–31. 10.1038/s41379-020-0624-y 32694616

[25] Dobrowolski J Pasca S Teodorescu P Selicean C Rus I Zdrenghea M. Persistent Basophilia may Suggest an “Accelerated Phase” in the Evolution of CALR-Positive Primary Myelofibrosis Toward Acute Myeloid Leukemia. Front Oncol (2019) 9:872. 10.3389/fonc.2019.00872 31555600 PMC6742718

[26] Park S Huh HJ Mun YC Seong CM Chung WS Chung HS. Calreticulin mRNA Expression and Clinicopathological Characteristics in Acute Myeloid Leukemia. Cancer Genet (2015) 208(12):630–5. 10.1016/j.cancergen.2015.11.001 26640226

[27] Burman J Svensson E Fransson M Loskog AS Zetterberg H Raininko R. The Cerebrospinal Fluid Cytokine Signature of Multiple Sclerosis: A Homogenous Response That Does Not Conform to the Th1/Th2/Th17 Convention. J Neuroimmunol (2014) 277(1-2):153–9. 10.1016/j.jneuroim.2014.10.005 25457841

[28] Yazdani Z Mousavi Z Ghasemimehr N Kalantary Khandany B Nikbakht R Jafari E. Differential Regulatory Effects of Chemotherapeutic Protocol on CCL3_CCL4_CCL5/CCR5 Axes in Acute Myeloid Leukemia Patients With Monocytic Lineage. Life Sci (2020) 240:117071. 10.1016/j.lfs.2019.117071 31783051

[29] Jeong KH Kim SK Seo JK Shin MK Lee MH. Association of GZMB Polymorphisms and Susceptibility to non-Segmental Vitiligo in a Korean Population. Sci Rep (2021) 11(1):397. 10.1038/s41598-020-79705-0 33431938 PMC7801456

[30] Šestáková Š Krejčík Z Folta A Cerovská E Šálek C Merkerová MD. DNA Methylation and Hydroxymethylation Patterns in Acute Myeloid Leukemia Patients With Mutations in DNMT3A and IDH1/2 and Their Combinations. Cancer Biomarkers Section A Dis Markers (2019) 25(1):43–51. 10.3233/CBM-182176 PMC13082415 30988238

[31] Austin R Smyth MJ Lane SW. Harnessing the Immune System in Acute Myeloid Leukaemia. Crit Rev Oncol Hematol (2016) 103:62–77. 10.1016/j.critrevonc.2016.04.020 27247119

[32] Paczulla AM Rothfelder K Raffel S Konantz M Steinbacher J Wang H. Absence of NKG2D Ligands Defines Leukaemia Stem Cells and Mediates Their Immune Evasion. Nature (2019) 572(7768):254–9. 10.1038/s41586-019-1410-1 PMC6934414 31316209

[33] He X Feng Z Ma J Ling S Cao Y Gurung B. Bispecific and Split CAR T Cells Targeting CD13 and TIM3 Eradicate Acute Myeloid Leukemia. Blood (2020) 135(10):713–23. 10.1182/blood.2019002779 PMC7059518 31951650

[34] Wang C Sun C Li M Xia B Wang Y Zhang L. Novel Fully Human anti-CD47 Antibodies Stimulate Phagocytosis and Promote Elimination of AML Cells. J Cell Physiol (2020) 236:4470–81. 10.1002/jcp.30163 33206395

