# Supplementary figures and images for: Development and Validation of a Novel Prognostic Model for Acute Myeloid Leukemia Based on Immune-Related Genes
Source: Front Immunol. 2021 May 5;12:639634. doi: 10.3389/fimmu.2021.639634 (PMC8131848; doi:10.3389/fimmu.2021.639634)

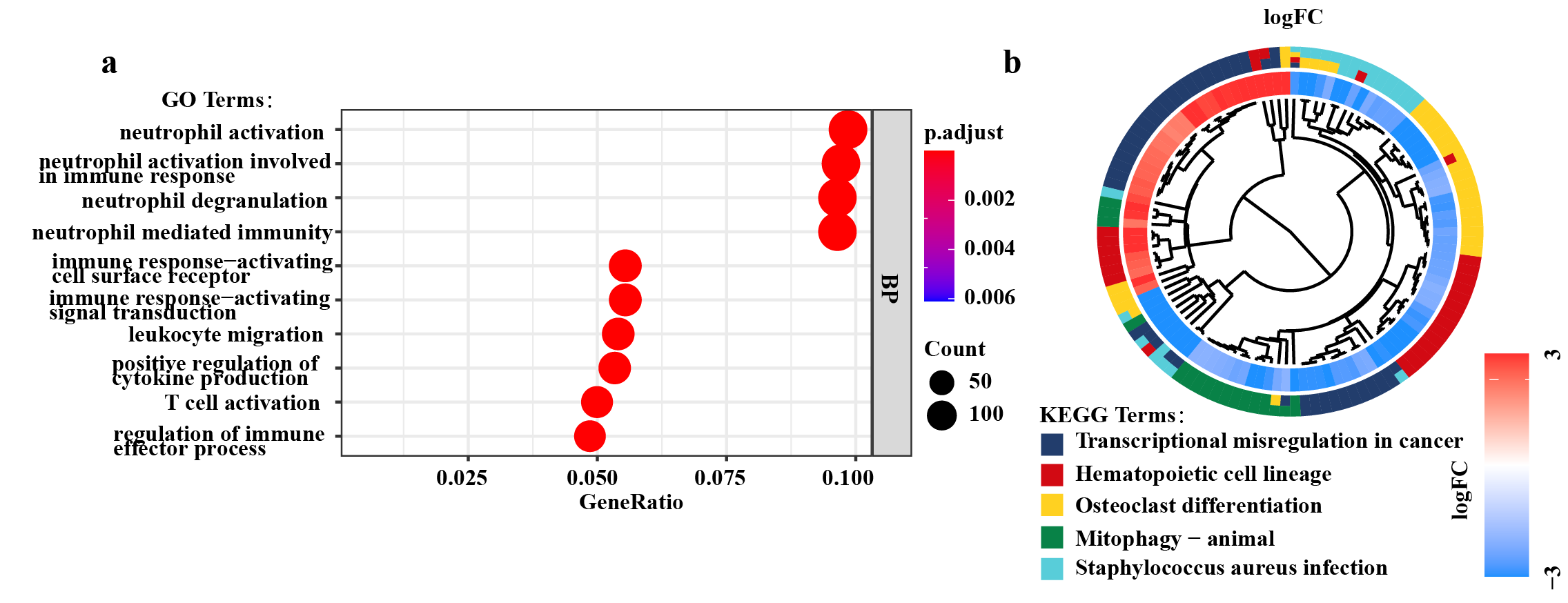

Supplement: Supplementary Figure 1 — Enrichment analysis of differentially expressed genes (DEGs) derived from AML patients and healthy individuals. (A) The top 10 Gene Ontology (GO) terms of biological process. (B) The top 5 Kyoto Encyclopedia of Genes and Genomes (KEGG) terms. [file Image_1.tif]

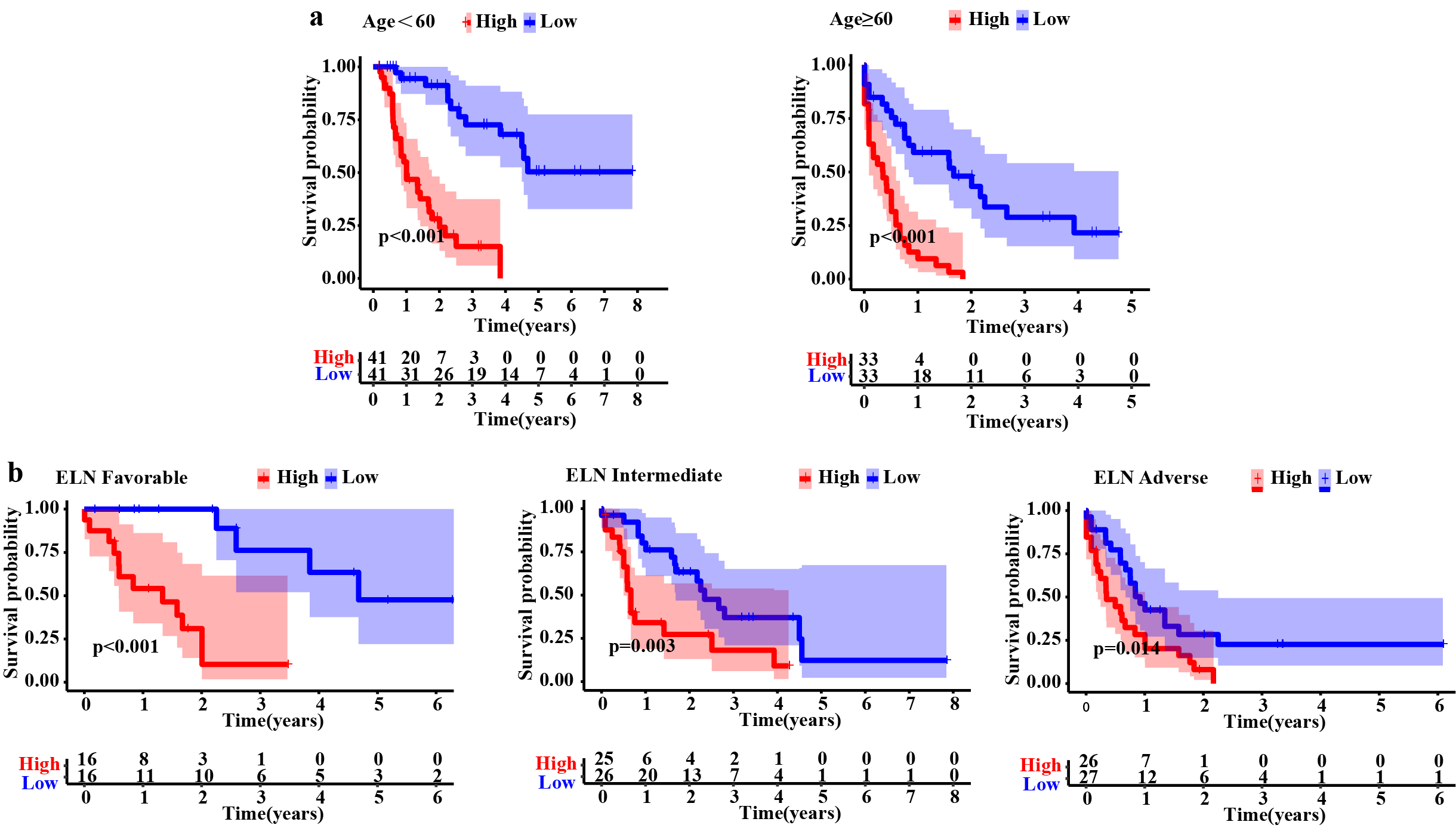

Supplement: Supplementary Figure 2 — Stratification analysis. Kaplan-Meier analysis of AML patients from TCGA-LAML cohort stratified by age (A) and ELN risk stratification system (B). [file Image_2.tif]
